# Comparative Analysis of Genetic Risk for Viral-Induced Axonal Loss in Genetically Diverse Mice

**DOI:** 10.3390/ijms262110727

**Published:** 2025-11-04

**Authors:** Tae Wook Kang, Aracely Perez-Gomez, Koedi Lawley, Colin R. Young, C. Jane Welsh, Candice L. Brinkmeyer-Langford

**Affiliations:** 1 Department of Veterinary Integrative Biosciences, College of Veterinary Medicine & Biomedical Sciences, Texas A&M University, College Station, TX 77843, USA; 2 Procter and Gamble Personal Health Care, Cincinnati, OH 45202, USA; 3 College of Veterinary Medicine and Biomedical Sciences, Colorado State University, Fort Collins, CO 80521, USA; 4 Department of Veterinary Pathobiology, College of Veterinary Medicine & Biomedical Sciences, Texas A&M University, College Station, TX 77843, USA; 5 School of Public Health, Texas A&M Health Science Center, College Station, TX 77843, USA

**Keywords:** Theiler’s murine encephalomyelitis virus (TMEV), Theiler’s virus-induced demyelination (TVID), Theiler’s virus-induced axonal loss (TVIAL), myelin loss, collaborative cross (CC) mouse strains, major histocompatibility complex (MHC), neuroimmune interactions

## Abstract

Among mouse models of neurological disease, Theiler’s murine encephalomyelitis virus (TMEV) provides a unique platform by using a naturally occurring viral trigger, paralleling the role of infections like Epstein–Barr virus in multiple sclerosis (MS). Just as not all individuals with predisposing viral infections develop the same neurological disease, not all mouse strains develop the same diseases following TMEV infection, so susceptibility is dictated by genetic background. For example, certain sets of alleles, called haplotypes, of the major histocompatibility complex (MHC) region have been associated with susceptibility to TMEV-induced demyelination (TVID) and MS. However, our previous work revealed that these MHC susceptibility haplotypes are not the sole contributors to TMEV-induced diseases in all mice. We infected mice from the genetically diverse Collaborative Cross (CC), a resource designed to reflect human population-level genetic variation. All 15 CC strains tested exhibited some form of neurological phenotype or CNS lesion following TMEV infection. However, chronic radiculoneuropathy characterized by axonal degeneration with myelin loss was observed in the CNS of only two strains, CC002 and CC023, which had markedly different immune responses and clinical profiles throughout the course of infection. Moreover, the pathology seen in CC002 and CC023 was not the same as what is typically seen in TVID. We used previous results from RNA sequencing of the hippocampus and spinal cord to test our hypothesis that myelin loss in these strains resulted from the convergent biological effects of multiple genetic risk variants, many previously unassociated with TMEV-induced diseases. These findings identify novel genetic targets and demonstrate the utility of genetically diverse models for uncovering complex neuroimmune interactions.

## 1. Introduction

Among mouse models of neurological disease, models using Theiler’s murine encephalomyelitis virus (TMEV) provide a unique perspective in that a naturally occurring infectious agent is the primary precipitating factor. TMEV infection captures the etiology of multiple sclerosis (MS) in people who have had prior viral infections, such as Epstein–Barr virus [1], human herpesvirus 6 [2], possibly herpes zoster [3], and human endogenous retroviruses [4,5]. For these individuals, “molecular mimicry” may cause the immune response to become sensitized to myelin proteins with similar structures to viral proteins. However, not all MS patients have experienced a predisposing virus, and not all people who have these viral infections ultimately develop demyelinating disease. For TMEV infection, the genetic background influences susceptibility to different neuropathologies [6,7,8,9,10,11]. The SJL/J mouse strain develops demyelinating disease after infection with TMEV [12,13], while the strain C57BL/6J develops epileptic seizures [14,15,16]. Because SJL/J and C57BL/6J mice are inbred, there is limited genetic diversity, which allows for consistent and reliable TMEV infection outcomes.

The TMEV infection model provides a perspective of MS that is both natural and human-relevant. Antecedent Epstein–Barr virus (EBV) infection has been linked to MS in humans [1,5,17,18,19]. Furthermore, as with TMEV infection, not all humans infected with EBV are equally susceptible to developing MS later on. In fact, recent findings revealed certain alleles of the EBV nuclear antigen 2 viral transcriptional regulator that are associated with MS risk [20,21]. Also, like TMEV, alleles of loci in the major histocompatibility complex are linked to MS [22,23,24], as well as to EBV [1,17,25,26].

Recently, we infected mice of the genetically diverse Collaborative Cross (CC) mouse resource with TMEV to evaluate outcomes of TMEV infection in backgrounds with significantly more genetic variation [27,28,29,30]. The CC was designed to mimic the type of genetic variation seen in human populations, thus distinguishing CC mouse strains from inbred strains, which have relatively less genetic variation [31]. Not all TMEV-infected CC mouse strains developed demyelination. In fact, none showed the same type of demyelinating disease as seen in SJL/J mice, though some strains showed evidence of axonal degeneration and myelin loss [28]. Therefore, in this study, we sought to determine the genetic factors underlying the occurrence of lesions that exhibited axonal degeneration and myelin loss as opposed to lesions without these pathologies. Here, we made use of histological and genetic data collected for our prior studies [27,28,32] and evaluated the findings with the aim of discriminating susceptibility to axonal degeneration and myelin loss regardless of the presence of other lesions.

## 2. Results

### 2.1. Lesion Burden Varied by Strain and Region, with Axonal Degeneration Only Found in Two Strains

**Figure 1:**
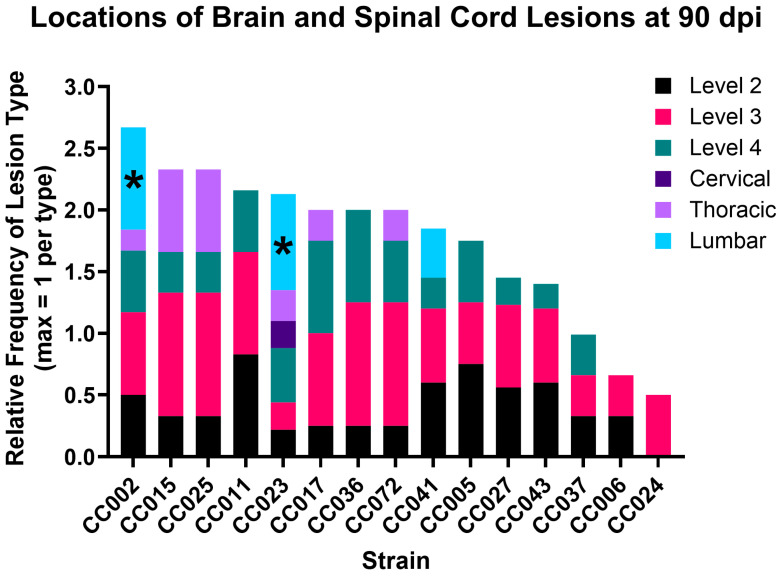
TMEV-induced lesion burden varied by CC strain, but lesions with myelin loss were only found in the lumbar region of CC002 and CC023 [28]. Each bar represents a strain and is divided by color to show lesion locations. The height of each colored segment reflects the proportion of mice with lesions in that region. Lesions with axonal degeneration, as described previously [28], are indicated by *. Levels 2, 3, and 4 are regions of the brain as defined previously [28]; cervical, thoracic, and lumbar are regions of the spinal cord.

We previously described lesion location and burden in CC mouse strains [28], and in that study, we focused particularly on the types of lesions found in six CC strains following TMEV infection. For the current study, we expanded this dataset to include lesion data for 15 CC strains (Table 1). All mouse strains included in the present study had lesions observed in at least one region in the brain. Strains CC015, CC017, CC025, and CC041 also had lesions in the spinal cord. CC002 had lesions in all regions examined except for the cervical spinal cord; CC023 also had lesions in all regions and was the only strain to have lesions in the cervical spinal cord. We identified axonal damage with myelin loss in the lumbar spinal cords of only two CC strains: CC002 and CC023 [28]. Figure 1 provides a graphical comparison of lesion burden across the 15 strains.

### 2.2. Genetic Predisposition to Neurological Disease Was Not Solely Based on the H2 Haplotype

While none of the CC strains we evaluated thus far had the same TMEV-induced demyelination (TVID) as seen in SJL/J mice, we investigated the possibility of the H2 haplotype contributing to the neuropathological outcomes in TMEV-infected CC mice. TVID has been associated with haplotypes of H2, which is the major histocompatibility complex in mice [33,34], located on mouse chromosome 17 (MMU17). The Collaborative Cross strains in this study inherited the H2 region from different founder strains. The only founder strain that was previously studied in relation to TMEV infection is C57BL/6J, a strain that is considered resistant to TVID. C57BL/6J mice have the *b* haplotype for H2; therefore, H2^b^ has been considered TVID-resistant [6,33].

**Figure 2:**
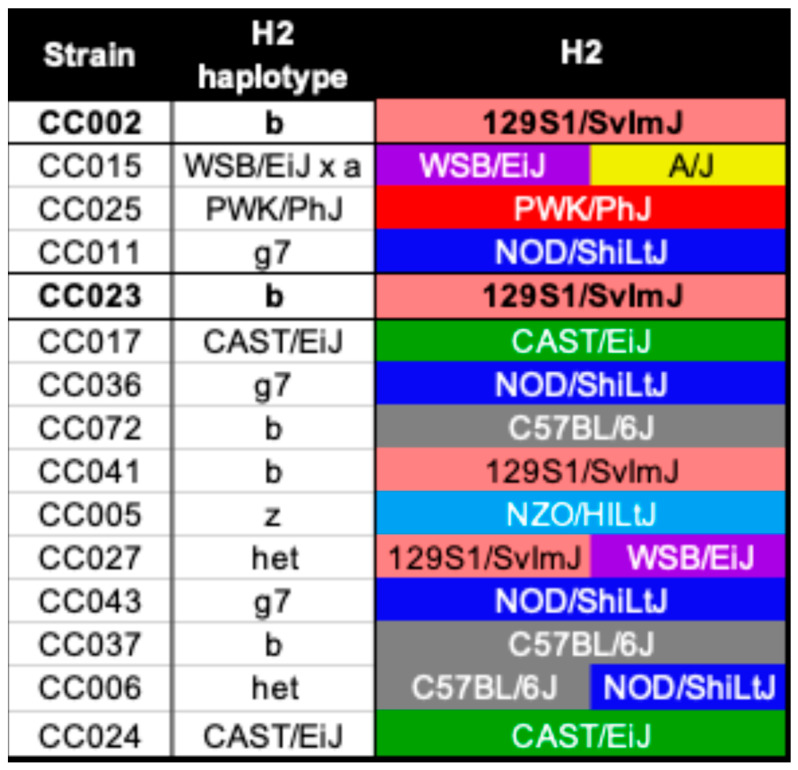
CC founder strain haplotypes for H2 and other known TVID risk loci. CC strains are listed in the same order as Figure 1. The H2 haplotype is in the second column. Each color in the other columns represents the CC founder strain from which each locus was inherited. Strains CC002 and CC023 have the same H2 haplotype, which was previously associated with resistance to TVID.

We identified the H2 haplotype for each of the CC strains in this study (Figure 2), finding that five strains had the resistant haplotype H2^b^. However, two of those five strains were CC002 and CC023, which developed axonal degeneration. CC002, CC023, and CC041 inherited the H2^b^ allele from the 129S1/SvImJ founder, while the other strains with H2^b^, CC037 and CC072, inherited it from C57BL/6J. Therefore, the H2^b^ haplotype inherited from C57BL/6J was associated with TMEV disease without axonal loss, but the haplotype itself was insufficient to confer protection from neuropathologies involving axon degeneration.

Other loci related to TMEV infection have been identified over the years. Some of these loci are specifically linked to TMEV persistence or demyelination in inbred mouse strains. Of note is a locus on mouse chromosome 10 (MMU10), which contains genes for interferon gamma (*Ifng*), interleukin 22 (*Il-22*), and a noncoding RNA known as Tmevp3 or NeST (for *nettoie Salmonella pas Theiler’s*, or “cleanup Salmonella not Theiler’s”) [35]. This region was inherited from different CC founder strains for CC002 and CC023, again showing that inheritance of the axonal degeneration phenotype is not simple.

### 2.3. QTL Associated with Lesion Locations Indicated That Genetic Background Was a Contributing Factor to Lesion Burden

We previously described lesion location and burden in CC mouse strains [28], with all histological evaluations performed by a pathologist blinded to strain identity. In that study, we focused particularly on the types of lesions found in six CC strains following TMEV infection. For the current study, we expanded this dataset to include lesion data for 15 CC strains, which allowed us to improve the detection of QTL associated with lesion location (Table 1). All mouse strains included in the present study had lesions observed in at least one region in the brain. Strains CC015, CC017, CC025, and CC041 also had lesions in the spinal cord. CC002 had lesions in all regions examined except for the cervical spinal cord; CC023 also had lesions in all regions and was the only strain to have lesions in the cervical spinal cord. We identified lesions in the lumbar spinal cords of only three CC strains: CC002, CC023, and CC041. CC002 and CC023 had axonal degeneration with myelin loss, distinguishing them from the other strains. This phenotype was most like the TVID phenotype of SJL/J mice among all CC strains included in this study; however, there were multiple differences that led us to distinguish the phenotypes of CC002 and CC023 from TVID.

**Figure 3:**
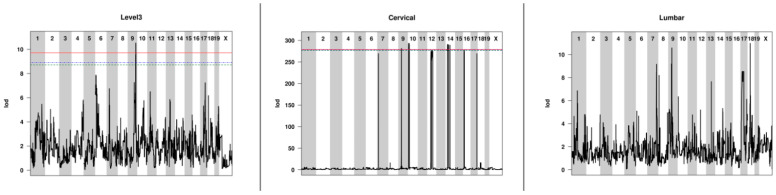
Manhattan plots for the level 3, cervical, and lumbar QTL. The lines at the tops of the level 3 and cervical plots show 85 (green dashed line), 90 (blue dotted line), and 95% (red line) significance threshold levels. There are no lines for the lumbar QTL plot because the QTL were not quite significant.

Lumbar spinal cord lesions were associated with SNP JAX00171047 on mouse chromosome 9 (MMU9; including 193 genes between 43.94 and 9.54 Mb) and SNP UNC29023821 on MMU18 (including 4 genes between 34.12 and 34.18 Mb); both associations were strong but not statistically significant. Significant QTL were identified for lesions located at brain level 3 (UNC17077443 on MMU9, including 127 genes between 103.72 and 107.51 Mb) and the cervical spinal cord (UNC16238010 on MMU9, including 11 genes between 41.80 and 42.31 Mb, UNC17405188 on MMU10, including 104 genes between 9.79 and 16.35 Mb, UNC23765091 on MMU14, including 1187 genes between 25.79 and 57.50 Mb, and UNC26402456 on MMU16, including 116 genes between 12.66 and 17.16 Mb). These QTL are summarized in Table 2 Manhattan plots for these QTL are shown in Figure 3, and allele plots for these QTL are found in Supplementary Figure S1.

### 2.4. Axonal Degeneration in CC Strains Differed from TVID Typically Associated with Inbred Mice

Demyelinated lesions in SJL/J and other TVID-susceptible inbred mouse strains have been associated with progressive demyelinating leukomyelitis [36,37,38,39]. The axonal degeneration aspect of TVID has been proposed to start with an inside-out mechanism: neurofilament accumulates as a result of the viral infection itself, which causes damage to the axon. An outside-in mechanism occurs next, wherein demyelination leads to the loss of glia and resulting axon vulnerability [38,40,41]. In both MS and TVID, damage includes inflammation, astrogliosis, demyelination, and axonal generation [42]. Cytokines, chemokines, and cells of the innate immune system are involved in the immune response against TMEV, which contributes to TVID susceptibility [43] along with variation in the proinflammatory Th17-mediated vs. antiviral Th1-mediated responses [44,45].

We previously found that the immune responses of TMEV-infected CC strains were not like those typically seen in TMEV-infected SJL/J mice at 90 days post-infection. This included the proinflammatory environment seen in SJL/J mice, which differed from that seen in CC002 and CC023. In fact, CC023 showed evidence of a somewhat subdued response overall, compared to SJL/J and CC002, with cytokine and chemokine levels typically lower than the average value measured for all strains in the study [29]. Because the immune landscape differed so much between SJL/J, CC002, and CC023 at the same time point following TMEV infection, variation in the appearance of TVID is not unexpected. In fact, it is likely that the axonal degeneration and myelin loss of CC002 and CC023 did not arise in the same way as TVID in SJL/J mice. Therefore, for the purposes of this study, we use the term Theiler’s Virus-Induced Axonal Degeneration (TVIAL) for CC strains that developed some type of axonal degeneration and myelin loss (CC002 and CC023).

The QTL allele effects differed for TVIAL vs. non-TVIAL strains at most QTL. We identified the Collaborative Cross founder strains from which each strain inherited each QTL. TVIAL strains CC002 and CC023 did not inherit the same founder alleles for any of the significant QTL or lumbar QTL. However, the TVIAL strains did have alleles that were not shared by any non-TVIAL strain for these QTL. For example, CC002 had heterozygous genotypes for the QTL UNC17405188, UNC23765091, and UNC29023821; no other strain studied was heterozygous at these QTL. The founder alleles inherited by CC023 were different from all other strains for the QTL listed in Table 2. Alleles and allele effects plots for these QTL are found in Supplementary Figure S1.

**Table 2:** Significant QTL were identified for two lesion locations, and the lumbar lesion location QTL included here were nearly significant. SNP IDs are provided for these QTL, along with chromosome (Chr) and position on that chromosome in megabases (Pos [Mb]) and in centimorgans (Pos [cM]). LOD scores and *p*-values reflect the degree of confidence in the identified associations, while the % Var column shows the percentage of variation in each phenotype that could be explained by the QTL. Finally, proximal and distal boundaries for each QTL region are shown in the last two columns. Bolded *p*-values indicate QTL deemed statistically significant by the gQTL program.

Lesion Location	SNP ID	Chr	Pos (Mb)	Pos (cM)	LOD	*p* -Value	% Var	Proximal (Mb)	Distal (Mb)
Level 3	UNC17077443	9	106.56	50.69	10.52	**2.90 × 10^−8^**	93.23	103.85	107.63
Cervical	UNC16238010	9	41.92	18.35	281.49	**1.04 × 10^−275^**	100.00	41.90	42.40
Cervical	UNC17405188	10	9.94	0.77	293.27	**1.91 × 10^−287^**	100.00	9.91	16.47
Cervical	UNC23765091	14	29.12	7.06	290.82	**5.33 × 10^−285^**	100.00	25.79	57.26
Cervical	UNC26402456	16	16.96	6.57	277.77	**5.25 × 10^−272^**	100.00	12.84	17.34
Lumbar	JAX00171047	9	49.57	23.55	10.58	**2.57 × 10^−8^**	93.33	44.03	49.63
Lumbar	UNC29023821	18	34.05	12.68	10.98	**1.13 × 10^−8^**	93.97	33.99	34.05

Newly identified QTL do not overlap with other QTL previously identified for TMEV infection-related pathologies. Other groups have previously identified at least 11 QTL based on clinical signs of TVID, demyelination, or viral load (summarized by Brahic et al. [33]). These are not part of the H2 haplotypes associated with TMEV phenotypes and are not located on the same chromosome as the H2 region; therefore, they are referred to as “non-H2” QTL in the literature. Four of these non-H2 QTL are located on the same chromosomes as the QTL we identified in this study (Table 2). However, centimorgan (cM) positions published for these non-H2 QTL indicate they are not likely to be the same genetic marker as we describe in the present study. Furthermore, none of the QTL in this study were located on the same chromosome as H2, emphasizing the novelty of these QTL for TMEV-related disease. Supplementary Table S3 includes more details regarding the genes residing in these QTL, which are candidates for TVIAL-related roles, including gene expression levels.

### 2.5. Gene Expression Distinguished TVIAL-Affected Mouse Strains from Those Not Affected by TVIAL

We next sought to identify how the expression of genes, QTL or otherwise, varied in a way that differently affected the strains with and without axonal degeneration. Previously, we collected transcriptomic data from the hippocampus and spinal cord of mice of both sexes from the same CC strains for which histological and QTL data were collected. As a result, the RNAseq data are relevant to the tissues in which we observed lesions [28]. For the present study, we focused specifically on those strains found to have TVIAL versus those without axonal loss (non-TVIAL). We sought to identify common gene expression signatures distinguishing TVIAL from non-TVIAL mice. We evaluated TMEV infection-induced changes in gene expression in relation to gene expression in mice that were sham-infected from the same strain (control). We identified those genes with expression significantly different between infected and sham-infected controls.

**Figure 4:**
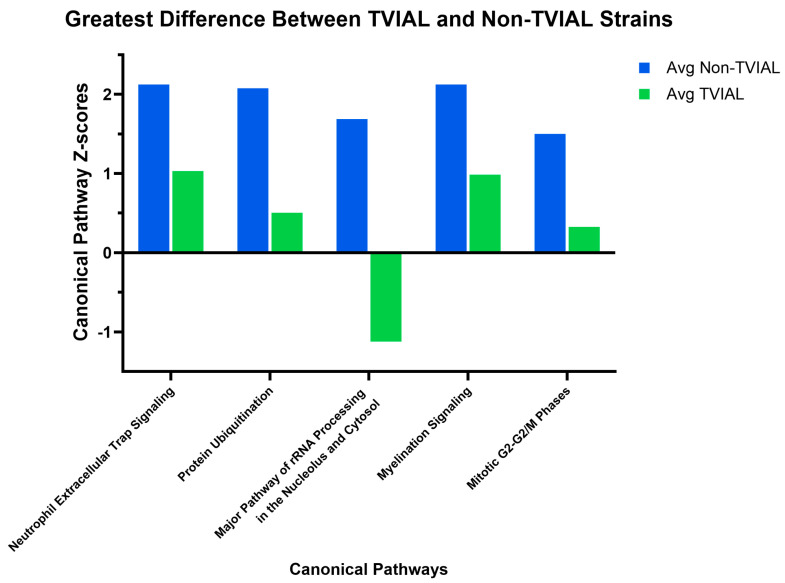
Canonical pathways with average Z-score differences of |Z| = 1.5 for TVIAL compared to non-TVIAL strains. Here, the pathway Z-scores were the most different between TMEV outcomes (e.g., average of all TVIAL vs. average of all non-TVIAL). Both TVIAL strains had similar Z-scores for these pathways. Blue bars represent average Z-scores for non-TVIAL strains, and green bars represent average Z-scores for TVIAL strains.

**Figure 5:**
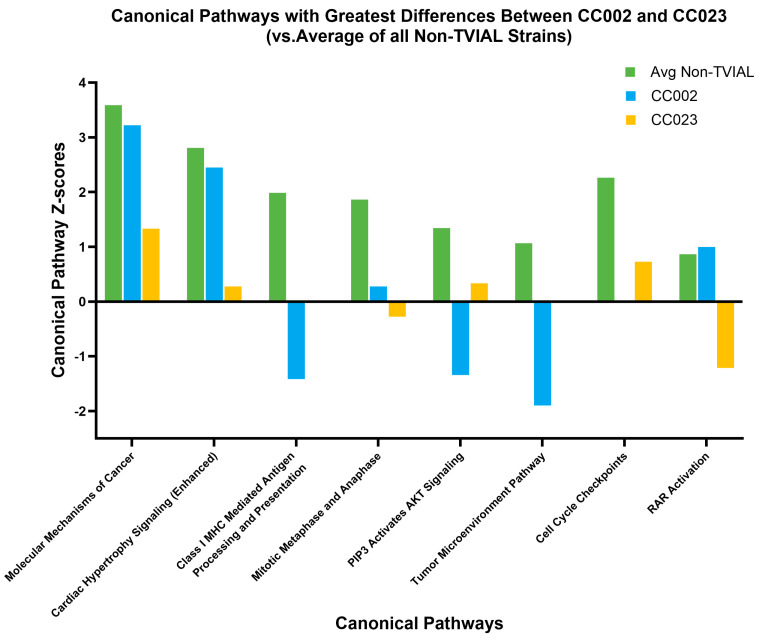
Canonical pathways with the greatest difference in scores between TVIAL strains (CC002 and CC023), where there was also a difference between each of the TVIAL strains and the combined average score of all non-TVIAL strains. Green bars represent average Z-scores for non-TVIAL strains, blue bars represent Z-scores for CC002, and gold bars represent Z-scores for CC023.

Differences in gene expression were associated with predicted activation or inhibition of specific canonical pathways, with patterns that varied between TVIAL and non-TVIAL strains. IPA canonical pathway analyses list pathways that are enriched for the dataset and scored based on *p*-values and percentage of overlapping genes versus the total number of genes in each pathway [46]. Therefore, this score represents the predicted activation (positive score) or inhibition (negative score) state of a given pathway. We identified 538 canonical pathways, and of these, we focused on the 34 pathways with scores for all 15 strains included in this study (Supplementary Table S1). Gene expression data for genes in each of the pathways shown in Figure 4 and Figure 5 are provided in Supplementary Table S2. Figure 4 shows the pathways with scores that varied by at least 2-fold between the collective average for all non-TVIAL strains compared to the collective average of the TVIAL strains. Both TVIAL strains had similar scores for these pathways.

Other canonical pathways further distinguished between TVIAL strains CC002 and CC023. Several pathways distinguished TVIAL strains CC002 and CC023 from each other, as well as from non-TVIAL strains (Figure 5). For the Mitotic Metaphase and Anaphase pathway and RAR Activation pathway, CC002 and CC023 had scores in opposing directions (i.e., activated vs. inhibited). In other cases, such as for the Class I MHC mediated antigen processing and presentation pathway, Tumor Microenvironment Pathway, and Cell Cycle Checkpoints pathway, CC002 or CC023 had a score of 0, indicating equal evidence for the pathway being activated and inhibited; scores of 0 are also included in Supplementary Table S1 for other pathways.

### 2.6. Biomarkers and QTL-Linked Genes Revealed a Regulatory Architecture Underlying TVIAL Susceptibility

We next turned our attention to the genetic relationships that contributed to the TVIAL vs. non-TVIAL outcomes. Accordingly, we investigated the roles of genes located within QTL regions. We identified 59 such genes with roles in canonical pathways, upstream regulators, and/or central drivers of causal networks. The genes, the QTL with which they are associated, and their role(s) and expression in non-TVIAL, TVIAL, CC002, and CC023 are summarized in Supplementary Table S3.

We identified gene members of canonical pathways that were located within QTL regions. The canonical pathway with the most QTL genes was RAR Activation, with four; Myelination Signaling, Molecular Mechanism of Cancer, and Protein Ubiquitination each had three. Of these, Myelination Signaling and Protein Ubiquitination were identified as varying between the TVIAL and non-TVIAL strains, while RAR Activation and Molecular Mechanism of Cancer were different between the individual TVIAL strains as well as the non-TVIAL strains. Next, 17 of the 59 QTL genes were identified as drivers of causal networks, including 4 genes that were found to drive two different causal networks each: *Il10ra*, *Il17rd*, *Ptgdr*, and *Rnf31*. Of the 43 genes identified as upstream regulators, one (*Ptgdr*) was found to regulate two pathways, and 13 genes were both upstream regulators and drivers of causal networks. Upstream regulators have direct impacts on downstream gene expression, while causal network drivers are affected by upstream regulation as part of multi-step causal networks. Genes identified as both types likely play central roles in their associated pathways. *Zbtb16*, specifically, is in the RAR Activation canonical pathway and was found to have roles as both an upstream regulator and a driver of causal networks; this cumulatively indicates an important role for *Zbtb16* in a pathway with distinct differences between CC002, CC023, and non-TVIAL strains.

Biomarkers for TVIAL strains included QTL genes. IPA defines biomarkers as molecules that are measurable and show consistent association with a specific biological state (such as phenotype), based on published experimental evidence. We identified known and novel molecular biomarker candidates for each CC strain using the Biomarker Filter Results feature of IPA, filtering by species (mouse), tissues (nervous system), and *p*-adj value equal to or less than 0.05. We then used the Biomarker Comparison Analyses feature to compare biomarker differences and similarities between non-TVIAL strains and TVIAL strains, as well as non-TVIAL and CC002 and CC023, and, finally, CC002 and CC023. From the resulting list of biomarkers, we identified 39 residing in QTL regions to identify those linked to strain-specific (CC002 or CC023) and/or shared (TVIAL) susceptibility. Shared TVIAL biomarkers represent a core susceptibility signature across strains with axonal loss. CC002-specific biomarkers suggest an inflammation-driven phenotype. In contrast, CC023-specific biomarkers were consistent with intrinsic neuronal and metabolic vulnerability. Together, these biomarker sets suggest that axonal loss in these strains may be influenced by the combined effects of multiple genetic variants, many not previously linked to demyelination or TVID (Supplementary Table S2). They provide novel candidate targets for future mechanistic studies and demonstrate the potential of genetically diverse mouse models for investigating complex neuroimmune interactions.

Several biomarker genes in QTL regions were also members of canonical pathways and/or predicted to be upstream regulators, suggesting potential mechanistic roles in the biological distinction between groups. TVIAL-specific biomarker *Ptger2*, CC002-specific biomarkers *Cbl*, *Cish*, and *Prkcd*, and CC023-specific biomarker *Abcc1* are predicted upstream regulators. *Cbl* is also a member of the Molecular Mechanisms of Cancer pathway, and *Prkcd* is part of the RAR Activation pathway. The RAR Activation pathway and Molecular Mechanisms of Cancer pathway were the most prominent of all canonical pathways in this study because each contained 3–4 QTL genes and one biomarker. The Z-scores of these pathways differed substantially between CC002 and CC023 (Figure 5). Both the Molecular Mechanisms of Cancer and RAR Activation pathways were activated in CC002, especially compared to CC023. In fact, the RAR Activation pathway was activated in CC002 and inhibited in CC023. CC002 had the highest level of expression for *Prkcd* out of all the strains evaluated, and the collective evidence suggests that *Cbl* and *Prkcd* may be mechanistically central to the phenotypic distinction between CC002, CC023, and other strains.

Genes with extreme expression values distinguished TVIAL from non-TVIAL strains and included biomarkers. We identified genes that had expression values in CC002 or CC023 that were either the highest or lowest of all the strains. To find potential novel genes relevant to TVIAL, we focused on this subset of genes with extreme expression values from CC002 and/or CC023 (Supplementary Table S3). None of these genes were located within QTL regions or canonical pathways, and none were upstream regulators or drivers of causal networks. However, seven were identified as having roles as biomarkers. The remaining genes either did not have an official known function or included part of H2, already well known to influence susceptibility to neurological outcomes following TMEV infection.

Among the significant differentially expressed genes listed in Supplementary Table S2, *H2-Bl*, *Ifit1bl1*, and *Iigp1* have immune-related functions [47,48]. *Chrna6* and *Slc6a3* have neural functions related to dopamine: *Chrna6* is associated with nicotine dependence but also affects the activity of dopaminergic neurons [49,50,51]; *Slc6a3* encodes the dopamine transporter and is involved in multiple neurological conditions ranging from autism to Parkinson’s disease [52]. *Zfp429* has a regulatory role [53]. The other genes are less well-characterized but may also play regulatory roles. For example, *Gm9796* is a long noncoding RNA (lncRNA) gene that is suspected to have roles in neurodegenerative diseases, specifically through the regulation of prohibitin [54,55,56,57]. *Irx2*, another gene listed, is a transcription factor that has been found to possibly serve as a biomarker for Parkinson’s disease [58,59]. Of the TVIAL-associated genes in Supplementary Table S2, *Eif3j2* is the only gene with extreme expression in CC023. This gene functions in the initiation of translation as part of the eukaryotic initiation factor 3 complex [60].

## 3. Discussion

In this study, we demonstrate that axonal damage and myelin loss following TMEV infection arise through genetically determined susceptibility, though not necessarily through the same routes. Previously, we collected transcriptomic data from hippocampal and spinal cord sections of mice from many of the same CC strains used for lesion and QTL identification in this study and at the same time point (90 days post-infection) [32]. We re-evaluated this transcriptomic dataset from the perspective of distinguishing key differences between CC strains that developed axonal loss (TVIAL) and those that did not develop TVIAL after infection with TMEV. Among the 15 Collaborative Cross (CC) mouse strains examined, only two—CC002 and CC023—developed definitive axonal degeneration, despite broad CNS lesion presence across all strains.

### 3.1. Polygenic QTL Signatures Underlie Lesion Susceptibility and Include Loci Not Previously Associated with TMEV Pathology

Haplotypes of the major histocompatibility complex (MHC) have been linked to demyelination after TMEV infection, along with a handful of other loci outside of the MHC. Both CC002 and CC023 carry the H2^b^ haplotype, found in classical inbred strains, such as C57BL/6J, which do not experience myelin loss, yet both developed TVIAL. These two CC strains inherited the H2^b^ haplotype from 129S1/SvImJ rather than C57BL/6J, demonstrating how genetic context and interactions with non-MHC loci override MHC haplotype-based predictions. To identify novel QTL associated with TVIAL, we performed a genome-wide QTL scan, which identified several loci associated with lesion burden, including near-significant associations for lumbar spinal cord lesions—the only site where unequivocal TVIAL was observed. The two TVIAL strains did not share founder alleles for any significant QTL, again showing how the axonal loss phenotype can be the product of different genetic architectures. This parallels the concept of genetic heterogeneity in complex human diseases, where distinct variant combinations can produce similar disease outcomes. The fact that none of the newly detected QTL overlap with previously described “non-H2” QTL underscores their novelty and potential importance in TMEV pathogenesis.

### 3.2. Canonical Pathway Divergence Between CC002 and CC023 Reflected Distinct Biological Strategies Leading to Axonal Degeneration

We revisited RNAseq data previously produced in our lab, this time with a focus on understanding how gene expression was differently affected between TVIAL and non-TVIAL mice. We contextualized these differences by comparing top canonical pathways, various regulatory molecules, and biomarkers to investigate how expression affected biological processes underlying axonal degeneration. We also evaluated differences in the two strains that developed TVIAL, i.e., CC002 and CC023, to improve our understanding of how different genetic variants can contribute convergently to TMEV-induced axonal loss.

Pathway-level analysis revealed two distinct patterns. First, for the TVIAL vs. non-TVIAL groups, pathways such as Myelination Signaling, Protein Ubiquitination, and Major Pathway of rRNA Processing in the Nucleolus and Cytosol showed clear separation, suggesting that both myelin repair processes and proteostatic control influence susceptibility to myelin loss. Second, several pathways, including RAR Activation and Molecular Mechanisms of Cancer, were differentially activated or inhibited between CC002 and CC023. This demonstrated mechanistic divergence, with CC002 showing signatures of inflammation-driven pathology and CC023 exhibiting features more consistent with intrinsic neuronal and metabolic vulnerability.

In contrast to CC023, CC002 showed similar activation states as non-TVIAL strains for the pathways Molecular Mechanisms of Cancer, Cardiac Hypertrophy Signaling, and RAR Activation. Activation of these pathways is correlated with cell growth and differentiation in different contexts. On the other hand, pathways that were strongly inhibited in CC002 but activated in non-TVIAL strains included Class I MHC-mediated antigen processing and presentation, PIP3 activates AKT signaling, and tumor microenvironment pathways. These pathways can affect immunity and cell cycle regulation. CC023 either did not show activation or inhibition of these pathways or showed a slight amount of activation. The most notable pathway for CC023 was RAR Activation, which was strongly inhibited compared to other strains. This could mean that cell growth and differentiation were impaired, perhaps negatively affecting remyelination pathways. CC023 mice had more clinical signs throughout the 90-day course of infection than CC002 mice, which could partially be attributed to reduced repair mechanisms. These canonical pathway differences suggest that axonal loss in each strain may result from distinct upstream triggers—such as heightened inflammatory signaling in CC002 versus impaired neuronal maintenance in CC023. Such divergent molecular routes to the same endpoint reinforce the relevance of approaches that consider genetic variation.

Genes associated with QTL within the Molecular Mechanisms of Cancer pathway showed expression differences that may have contributed to axonal degeneration in the TVIAL strains, as well as to differences between CC002 and CC023. One such gene, CBL, encodes an E3 ubiquitin ligase that negatively regulates several signaling pathways involved in cellular growth and regeneration [61]. Both TVIAL strains exhibited higher CBL expression than non-TVIAL strains, which could lead to premature suppression of trophic signaling and, ultimately, axonal degeneration. Among the TVIAL strains, CBL expression was highest in CC023, potentially contributing to greater degeneration in this strain.

Similarly, PRKDC, which encodes a DNA-dependent protein kinase catalytic subunit that promotes cell survival under oxidative stress, such as that caused by neuroinflammation [62], was elevated in both TVIAL strains relative to non-TVIAL strains. Although PRKDC activation normally supports survival under oxidative conditions, chronic activation, as observed in neurodegenerative diseases such as Alzheimer’s disease, can result in maladaptive signaling characterized by persistent inflammation and the accumulation of damaged cells [63]. CC023 displayed much higher PRKDC expression than CC002, suggesting a stronger shift toward this maladaptive state. WNT5A expression was downregulated in non-TVIAL strains but upregulated in both TVIAL strains, particularly in CC023. Aberrant WNT5A signaling has been linked to neurodegenerative processes, consistent with its expression pattern in these mice [64,65].

QTL-associated genes within the Retinoic Acid Receptor (RAR) Activation pathway included CHAT, DRD2, PRKCD, and ZBTB16. Compared with the average of all non-TVIAL strains, CC002 showed higher expression of CHAT, DRD2, and PRKCD and lower expression of ZBTB16. In contrast, CHAT expression in CC023 was similar to that of non-TVIAL strains, while DRD2, PRKCD, and ZBTB16 were altered in the same direction as in CC002 but with smaller differences relative to non-TVIAL strains. The RAR Activation pathway was strongly inhibited in CC023 compared with CC002 and non-TVIAL strains, suggesting that these QTL-associated genes did not function equivalently in the two TVIAL strains. Retinoic acid typically enhances CHAT expression and, in CC002 but not CC023, may represent a compensatory mechanism that mitigates TMEV-induced neuroinflammatory damage [66,67]. DRD2 modulates neuroinflammation and can exert either protective or neurotoxic effects depending on context [68,69]; CC002 may have derived greater benefit from these effects than CC023. PRKCD responds to oxidative cues and can be either pro-regenerative or prodegenerative depending on the inflammatory environment [70,71,72,73]. Similarly, ZBTB16 expression is influenced by stress conditions and may promote or suppress regeneration and apoptosis [74].

Together, these findings suggest that both the Molecular Mechanisms of Cancer and RAR Activation pathways reflect a shared imbalance between regenerative and stress-response signaling in the TVIAL strains. In CC002, partial preservation of RAR-driven trophic signaling, including higher CHAT and DRD2 expression, may have counteracted the detrimental effects of elevated CBL and PRKDC, thereby supporting limited repair. In contrast, CC023 appeared to have a more profound suppression of RAR-mediated protective programs alongside exaggerated activation of stress and inflammatory pathways, a combination that may accelerate axonal degeneration and impair recovery.

### 3.3. Biomarker Integration Suggested Core and Strain-Specific Susceptibility Signatures

Our biomarker analysis identified both shared TVIAL signatures and strain-specific markers linked to QTL regions. Shared biomarkers likely represent core contributors to susceptibility to axonal loss, whereas CC002-specific markers (e.g., *Cbl*, *Prkcd*) and CC023-specific markers (e.g., *Abcc1*) may underlie the strains’ distinct clinical and pathological profiles. The RAR Activation and Molecular Mechanisms of Cancer pathways each contained multiple QTL genes and biomarkers, and RAR Activation had opposing activation states between CC002 and CC023. This suggests that pathway-level directionality, not just the presence or absence of QTL genes/biomarkers, is crucial to determining variations in the axonal degeneration outcome.

We also identified seven biomarker genes with expression values that were the highest or lowest across all 15 strains (Supplementary Table S3). These genes were not part of canonical pathways, QTL regions, or predicted upstream regulators. Despite this, their known functions suggest plausible mechanistic relevance to TVIAL. Genes with the highest expression in all strains, and identified as biomarkers for TVIAL, included *Chrna6* (α6 nicotinic acetylcholine receptor subunit) and *Slc6a3* (dopamine transporter). The functions of these genes implicate altered dopaminergic signaling, which can modulate microglial activation, T cell function, and oligodendrocyte biology, as a novel key factor affecting TVIAL biology. Two other TVIAL biomarkers with extremely high expression were *Irx2* (transcription factor) and *Zfp429* (KRAB zinc-finger protein); these genes may influence neural identity programs and antiviral transcriptional control, respectively. Biomarkers specific for CC002, which also had the highest expression in all strains, were *Ifit1bl1* (IFN-induced protein) and *Iigp1* (immunity-related GTPase). The functions of these two genes point to a heightened type I interferon and innate immune response for CC002 mice. This finding supports the earlier conclusion related to top canonical pathways activated or inhibited in CC002, namely, that inflammation played a more important role in CC002 pathology than in CC023. Additionally, we previously measured cytokine levels at the acute and chronic phases of TMEV infection, which indicated greater inflammation in CC023 compared to CC002 [29,30]. On the other hand, *Eif3j2* (translation initiation factor) was the sole biomarker gene with extreme expression in CC023, and in this case, expression was the lowest of all strains. The function of *Eif3j2* suggests possible constraints to protein synthesis in CC023 mice, potentially limiting repair responses despite preserved antiviral capability. *Eif3j2* is part of the translation initiation complex eIF3, which is documented to be dysregulated in neurological disorders, including epilepsy and Parkinson’s disease [75,76]. In summary, the combination of biomarkers with functions related to neural signaling, innate immune effectors, and translational machinery is consistent with the idea that TVIAL susceptibility arises from the convergence of multiple, independently variable systems. For the TVIAL strains described here, these systems affect neuronal excitability, immune response, and repair capacity.

### 3.4. Different Genetic Signatures Underlying Axonal Loss Models Variation Seen in Human MS

Multiple sclerosis varies in prognosis and presentation across populations, sexes, ages, and more [77,78,79,80,81,82,83]. It is therefore helpful that there are different animal models of MS, such as the experimental autoimmune encephalitis model [84,85,86], the cuprizone model [87], the lysolecithin model [88], and the viral infection model using TMEV [89,90]. Each of these models enables the study of different aspects of MS, such as relapsing–remitting vs. primary progressive forms of MS. However, the mice used typically must be from inbred strains to consistently reproduce the phenotypes being evaluated. In our work, we focused on the roles of genetic variation on disease presentation, using the TMEV infection model to replicate the relationships between antecedent viral infection and MS, such as seen for Epstein–Barr infection [1,5,17,18,19]. We used the Collaborative Cross recombinant–inbred panel of mouse strains to represent the level of genetic diversity found in different humans [28,30,31,91,92,93,94]. By doing so, we were able to capture a variety of neurological outcomes to the viral infection and identify which ones were most relevant to MS pathology. More importantly, we observed two CC strains with different genetic contributors to the demyelinating disease that affected each strain. We described this disease here as axonal loss to distinguish the appearance of demyelination in these CC strains from that seen more often in the TMEV-infected SJL/J inbred mouse strain, which has been studied for decades as a model for MS [8,9,37,95,96,97,98,99]. Accordingly, we postulate that TMEV-infected CC mouse strains CC002 and CC023 are novel animal models of MS with context specificity. Such models will be valuable for the development of nuanced treatments that can be customized for different patients.

### 3.5. Limitations and Future Directions

This study was limited by sample size and by the inability to assess cell-type-specific gene expression or sex-stratified effects. More mice from both sexes could help identify sex-related phenotypes and gene expression profiles. Future work involving single-cell transcriptomics and epigenetic profiling could help refine the cellular pathways driving axonal degeneration and myelin loss, while functional studies of key candidates, such as *Prkcd* and *Abcc1*, are needed to validate their causal roles. Such functional studies may include knockout or overexpression studies. Single-cell level longitudinal analyses of disease progression may also help define early versus late drivers of degeneration and myelin loss and reveal mechanisms specific to different cell types that vary based on genetic background. Additionally, future work could incorporate other environmental exposures along with viral infection to better understand how multiple factors shape TMEV outcomes.

## 4. Materials and Methods

### 4.1. Mice

All animal care protocols were in accordance with NIH Guidelines for Care and Use of Laboratory Animals and were approved by the Texas A&M University Laboratory Animal Care and Use Committee under animal use protocol numbers 2017-0082 (approved 20 July 2017) and 2020-0065 (approved 21 May 2020). The mice used for this study were previously used in other studies by our lab [28,32]. The mice were housed 4–5 to a cage in an AAALAC-approved facility under a 14 h light and 10 h dark cycle with *ad libitum* food and water. Breeding of all CC mice was performed in-house at Texas A&M University. Of the 151 mice used originally for RNA sequencing, 68 infected mice were included in the present study, collectively representing 31 females and 37 males from 15 CC strains (Table 3). The infected mice were anesthetized by isoflurane inhalation (MWU, Meridian, ID, USA) and intracerebrally injected into the right mid-parietal cortex (approximately 1.5 mm ventral) with 5.0 × 10^4^ plaque-forming units (PFUs) of the BeAn strain of TMEV (American Type Culture Collection [ATCC] VR 995, Manassas, VA, USA) in 20 µL of phosphate-buffered saline (PBS). A total of 52 sham-infected mice from the same strains and sexes, including 26 females and 26 males, were similarly injected with PBS only. The mice were observed and weighed daily to monitor general health. Measures were taken to minimize mouse pain and stress, including the provision of softened food pellets. The mice that lost more than 20% of their pre-infection body weight prior to the 90 dpi endpoint were euthanized and excluded from this study. The mice were euthanized between 87 and 94 dpi via intraperitoneal (i.p.) injection of a lethal dose of Beuthanasia -D Special 150 mg/kg (Schering-Plough Animal Health - Merck and Co Inc., Rahway, NJ, USA), as previously described [100]. The mice were transcardially perfused through the left ventricle with 10 mL of ice-cold phosphate-buffered saline. Following perfusion, one cerebral hemisphere and the spinal cord were collected and fixed for at least 48 h in 10% formalin.

### 4.2. RNA Sequencing Dataset

RNA sequencing (RNAseq) data were collected from the hippocampus and thoracic spinal cord 90 days after TMEV infection, as reported previously [32]. RNA samples isolated from the hippocampus and spinal cords of the Collaborative Cross mouse strains were quantified with a Qubit Fluorometer (Life Technologies, Carlsbad, CA, USA) with a broad-range RNA assay, and concentrations were normalized for library preparation. RNA quality was verified on an Agilent TapeStation with an RNA ScreenTape. Messenger RNA sequencing libraries were prepared using an Illumina TruSeq Stranded mRNA preparation kit (Illumina, San Diego, CA, USA). Barcoded libraries were pooled at equimolar concentrations and sequenced on an Illumina NovaSeq 6000 S4 150-cycle paired-end sequencing kit. Sequencing FASTQ files were generated by Illumina BaseSpace using the bcltofastq program.

A total of approximately 9.6 billion paired-end reads from the mice were checked to trim any adapter sequences and low-quality bases using Trimmomatic [101], resulting in approximately 8.6 billion filtered reads (89.3%), out of which a total of 7.9 billion filtered reads (approximately 92%) mapped to the combined reference sequences of *Mus musculus* (mm10 ENSEMBL release 99) genome assembly and the TMEV virus reference sequence from NCBI (https://www.ncbi.nlm.nih.gov/nuccore/M16020.1/ accessed 1 May 2021). Read mapping was performed using STAR [102]. Transcript-wise counts were generated using the featureCounts tool from the SUBREAD package [103]. Differential gene expression tests were then performed using DESeq2 [104] following the recommended guidelines by the authors. Gene expression values were normalized using TMEV-infected and sham-infected mice from the same sex and strain.

As described in study [32], further evaluation was performed using Ingenuity Pathways Analysis (IPA) software version 150276282 [105] to evaluate gene expression data. IPA identified key networks and pathways for each individual strain, for all non-TVIAL strains combined, and for the TVIAL strains combined. IPA calculates *p*-values differently depending on the analysis, as described [106]. We used the Ingenuity Knowledge Base (genes only) as the reference set. In IPA, the activation/inhibition states of canonical pathways are predicted based on a z-score algorithm, taking into account the activation state of key molecules and the causal relationships between molecules. In general, significance was determined using Fisher’s Exact Test. We applied the Benjamini–Hochberg method for multiple testing correction when identifying significant canonical pathways, upstream regulators, networks, and diseases/functions. Biomarkers were identified for each response group using IPA’s Biomarker Filter function.

### 4.3. Histological Evaluation

In our previous work [27,28], coronal sections of the brains of all mice were collected at 4 different levels (as initially defined [107]): level 2 (frontoparietal cortex, septal nuclei and caudate–putamen, nucleus accumbens), level 3 (frontoparietal cortex, hippocampus [CA regions—1, 2, 3, dentate gyrus], and thalamus), level 4 (occipital cortex and rostral colliculi of the midbrain), and level 6 (cerebellum, cerebellar peduncles, and pons). Level 1 includes the olfactory bulb, and level 5 corresponds to the inferior colliculus. We did not evaluate these levels to the same extent as the others, and so they are not included in this report. Additionally, transverse sections were collected from the cervical, thoracic, and lumbar spinal cords. All sections were processed, embedded in paraffin wax, and stained with hematoxylin and eosin (H&E). All slides were reviewed by a board-certified veterinary pathologist, blinded to slide identity, using an Olympus BX43-F microscope at 40× magnification with a DP73 camera, ND filters, and CellSens v.4.4 Standard Software. All sections of the brain were also scanned at 20× and viewed using ImageScope (Aperio Technologies, Vista, CA, USA) to map, trace, and transpose onto schematics using the Allen Mouse Brain Atlas, as well as Adobe Illustrator v.21 (Adobe Systems) for lesion location comparison within and across mouse strains. All histological evaluations were performed by an experimenter blinded to the treatment of the animals.

### 4.4. Association of Quantitative Trait Loci (QTL) with Lesion Burden

To quantify lesion burden for each strain of mice, we counted the number of mice with a lesion present at a given location, divided by the total number of infected mice. We then associated the calculated lesion burden with specific genomic regions using the gQTL online software platform [108], which enabled QTL identification within the genomes of the CC mice strains included in this study. Significance thresholds were determined using 1000 permutations and a *p*-value of less than 0.05. The genetic complement of the QTL regions was further evaluated using Ensembl BioMart [109]. We identified genes located in the QTL regions, along with their respective gene ontology (GO) terms and descriptions of phenotypes previously associated with those genes.

## 5. Conclusions

This study showed that TMEV-induced axonal loss (TVIAL) in genetically diverse mice is complex and heterogeneous, arising from the convergent effects of multiple genetic variants rather than the classic TVID pathway, and that genetically diverse models are essential for uncovering these neuroimmune mechanisms and therapeutic targets. This information is valuable because of its relevance to the level of human genetic diversity underlying complex neurological conditions such as MS.

## Figures and Tables

**Table 1:** The numbers indicate the percentage of all mice of a given strain with a lesion in the specified area. Levels 2, 3, and 4 are regions of the brain as defined previously [28]; cervical, thoracic, and lumbar are regions of the spinal cord.

Strain	Level 2	Level 3	Level 4	Cervical	Thoracic	Lumbar
CC002	50%	67%	50%	0%	17%	83%
CC005	75%	50%	50%	0%	0%	0%
CC006	33%	33%	0%	0%	0%	0%
CC011	83%	83%	50%	0%	0%	0%
CC015	33%	100%	33%	0%	67%	0%
CC017	25%	75%	75%	0%	25%	0%
CC023	22%	22%	44%	22%	25%	78%
CC024	0%	50%	0%	0%	0%	0%
CC025	33%	100%	33%	0%	67%	0%
CC027	56%	67%	22%	0%	0%	0%
CC036	25%	100%	75%	0%	0%	0%
CC037	33%	33%	33%	0%	0%	0%
CC041	60%	60%	25%	0%	0%	40%
CC043	60%	60%	20%	0%	0%	0%
CC072	50%	100%	0%	0%	0%	0%

**Table 3:** Numbers of female and male mice from 15 CC strains that were evaluated for lesion burden and location. Numbers of mice used for RNAseq are shown in parentheses [32].

Strain	TMEV-Infected F	TMEV-Infected M	Sham-Infected F	Sham-Infected M
CC002	4 (1)	3 (2)	3 (1)	3 (1)
CC005	2 (2)	2 (4)	2 (3)	2 (3)
CC006	2 (3)	1 (2)	1 (4)	1 (2)
CC011	2 (3)	4 (4)	2 (3)	2 (3)
CC015	1 (1)	2 (2)	2 (1)	1 (2)
CC017	3 (1)	1 (3)	1 (2)	2 (3)
CC023	5 (1)	4 (1)	4 (3)	3 (2)
CC024	1 (1)	1 (1)	1 (1)	1 (0)
CC025	3 (1)	2 (1)	1 (1)	0 (0)
CC027	4 (3)	5 (1)	5 (2)	5 (3)
CC036	1 (1)	3 (6)	1 (1)	1 (2)
CC037	2 (3)	1 (4)	2 (2)	2 (3)
CC041	0 (2)	4 (2)	0 (2)	2 (0)
CC043	1 (0)	2 (2)	1 (1)	1 (1)
CC072	0 (0)	2 (2)	0 (1)	0 (0)

## Data Availability

The original contributions presented in this study are included in this article/the Supplementary Materials. Further inquiries can be directed to the corresponding author.

## Supplementary Materials

The following supporting information can be downloaded at https://www.mdpi.com/article/10.3390/ijms262110727/s1.
