# Supplementary material for: Comparative Analysis of Genetic Risk for Viral-Induced Axonal Loss in Genetically Diverse Mice
Source: Int J Mol Sci. 2025 Nov 4;26(21):10727. doi: 10.3390/ijms262110727 (PMC12608172; doi:10.3390/ijms262110727)
Supplement: Supplementary file 1 [file ijms-26-10727-s001.zip › Supplementary Figure 1 - haplotypes for QTL.pptx]

## Slide 1
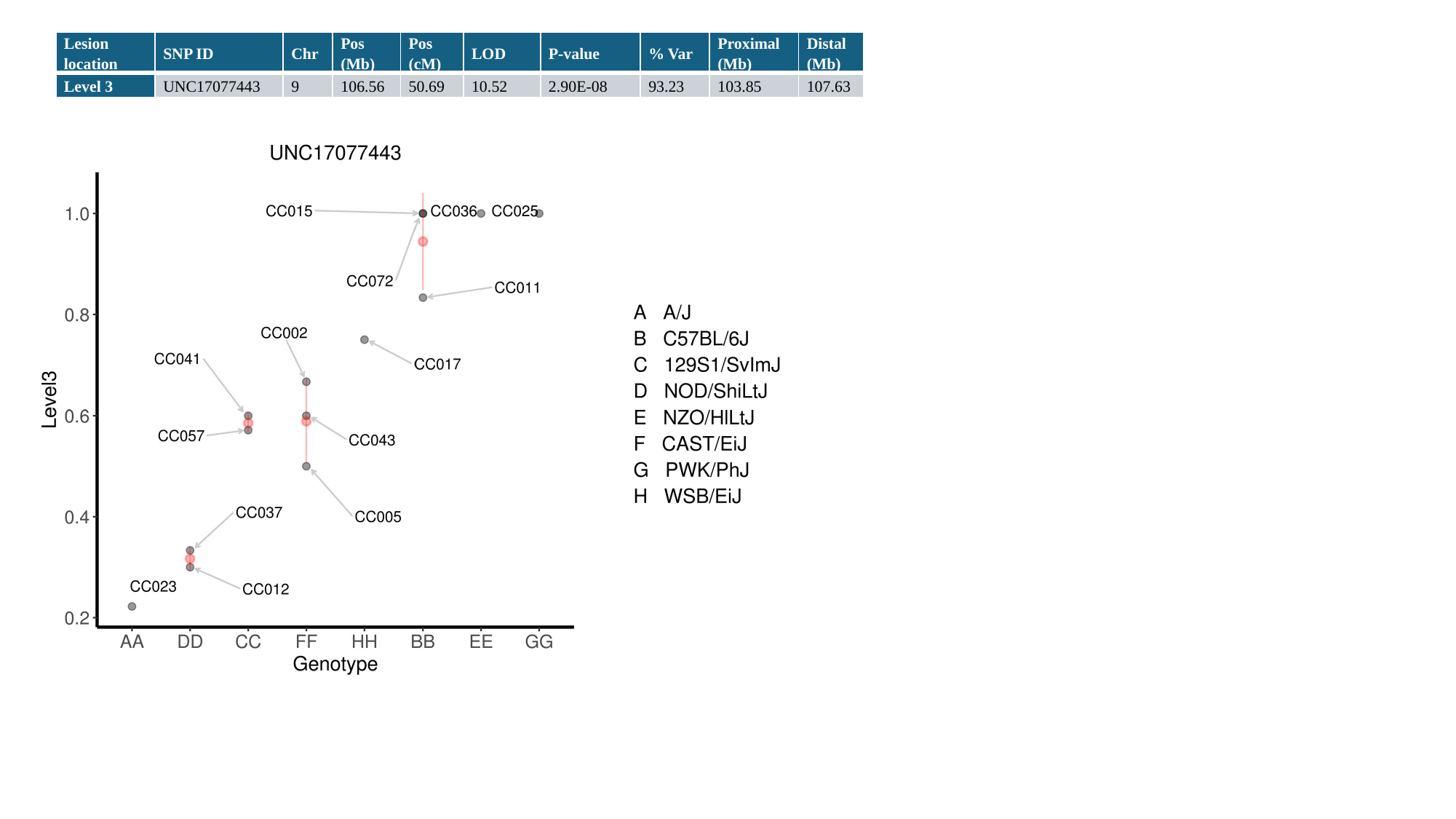

| Lesion location | SNP ID | Chr | Pos (Mb) | Pos (cM) | LOD | P-value | % Var | Proximal (Mb) | Distal (Mb) |
| --- | --- | --- | --- | --- | --- | --- | --- | --- | --- |
| Level 3 | UNC17077443 | 9 | 106.56 | 50.69 | 10.52 | 2.90E-08 | 93.23 | 103.85 | 107.63 |

## Slide 2
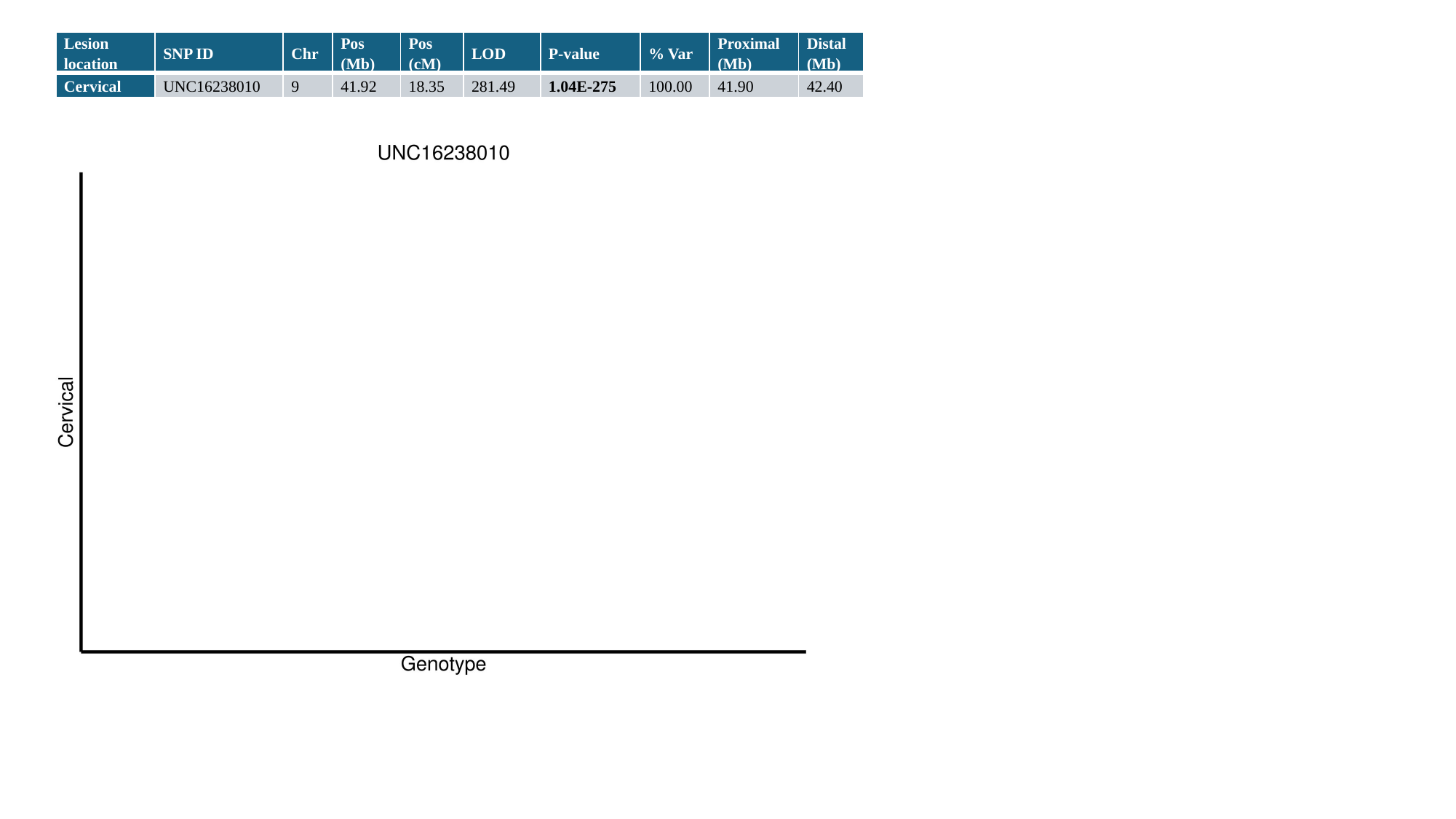

| Lesion location | SNP ID | Chr | Pos (Mb) | Pos (cM) | LOD | P-value | % Var | Proximal (Mb) | Distal (Mb) |
| --- | --- | --- | --- | --- | --- | --- | --- | --- | --- |
| Cervical | UNC16238010 | 9 | 41.92 | 18.35 | 281.49 | 1.04E-275 | 100.00 | 41.90 | 42.40 |

## Slide 3
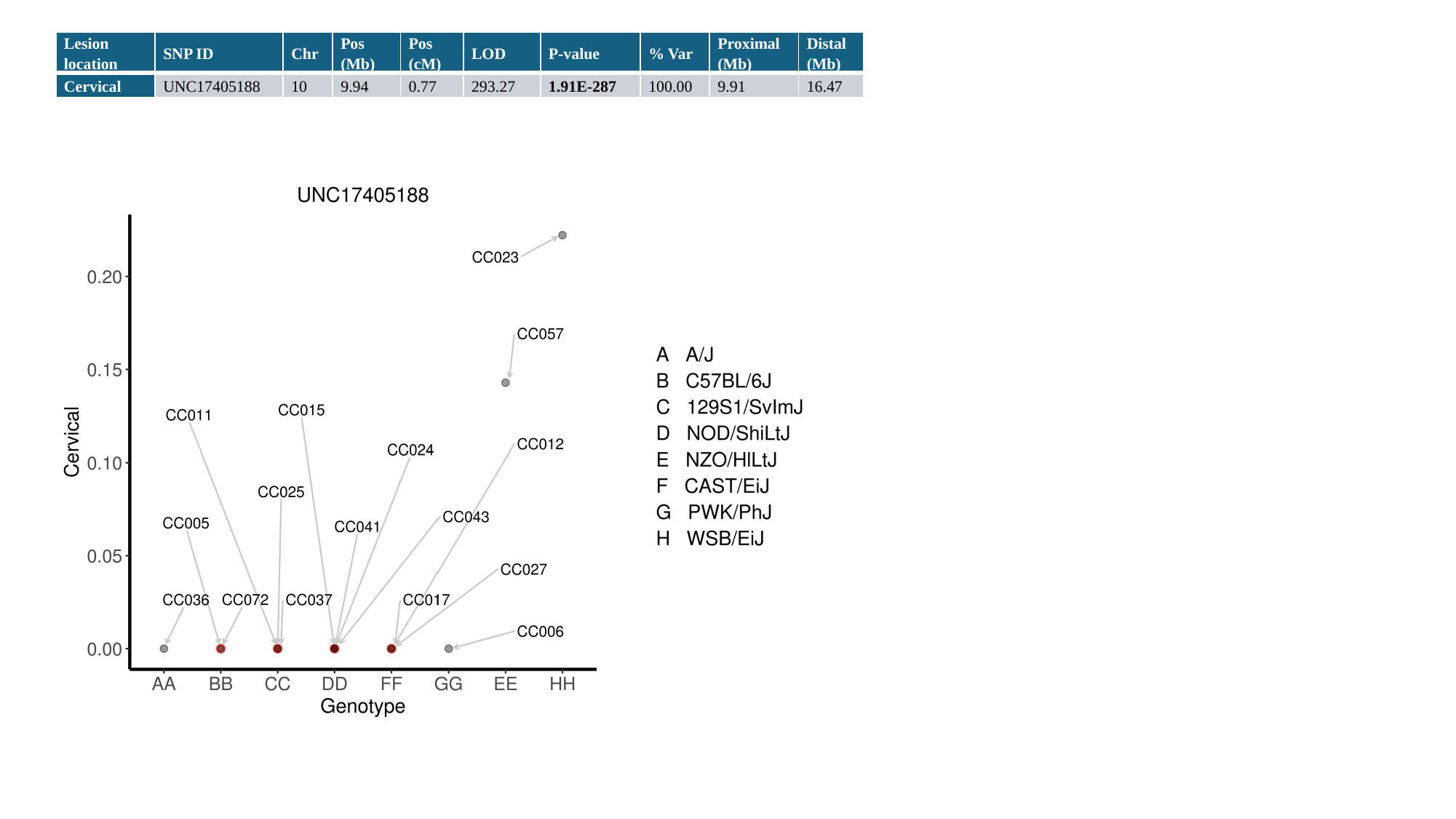

| Lesion location | SNP ID | Chr | Pos (Mb) | Pos (cM) | LOD | P-value | % Var | Proximal (Mb) | Distal (Mb) |
| --- | --- | --- | --- | --- | --- | --- | --- | --- | --- |
| Cervical | UNC17405188 | 10 | 9.94 | 0.77 | 293.27 | 1.91E-287 | 100.00 | 9.91 | 16.47 |

## Slide 4
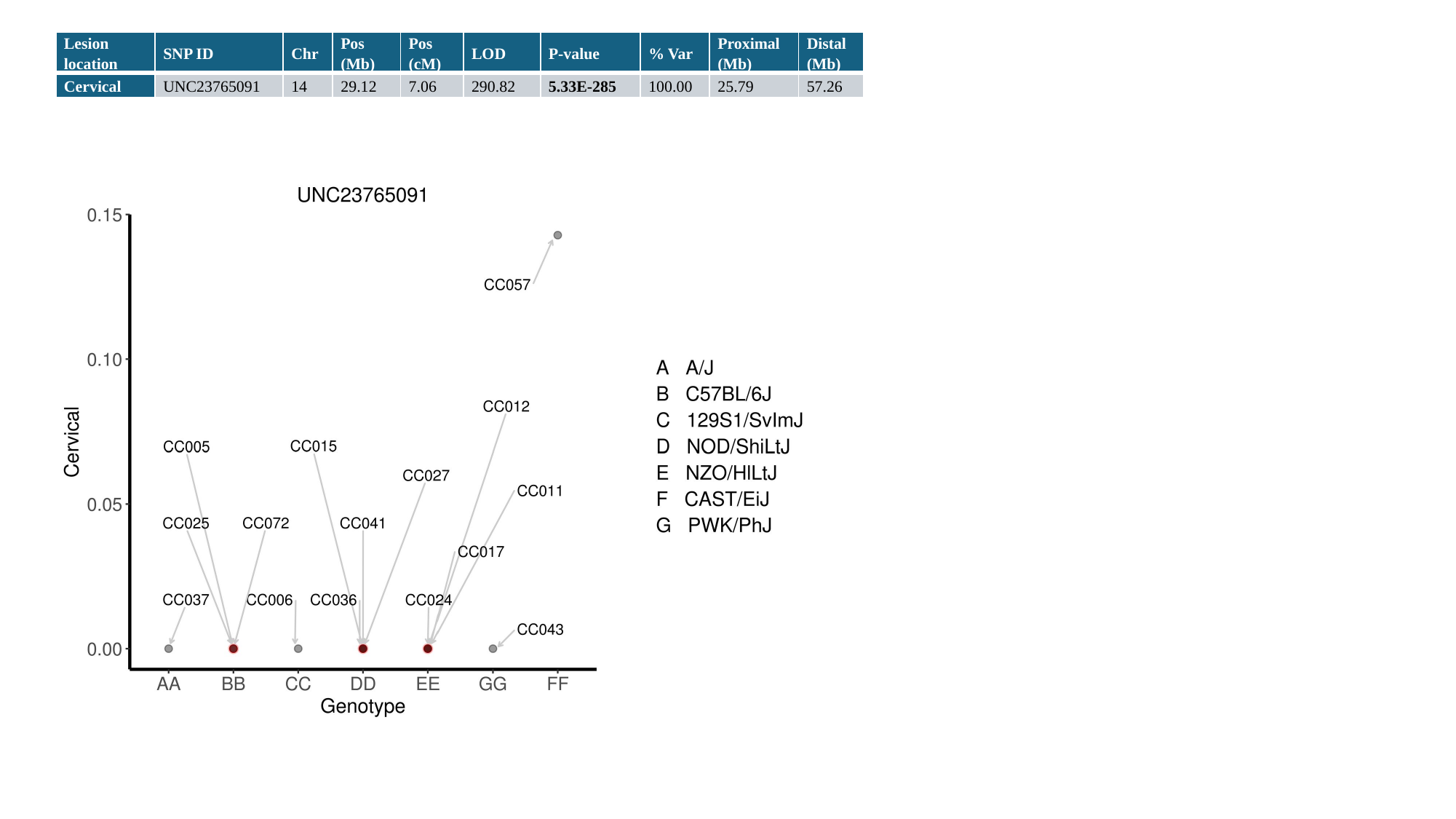

| Lesion location | SNP ID | Chr | Pos (Mb) | Pos (cM) | LOD | P-value | % Var | Proximal (Mb) | Distal (Mb) |
| --- | --- | --- | --- | --- | --- | --- | --- | --- | --- |
| Cervical | UNC23765091 | 14 | 29.12 | 7.06 | 290.82 | 5.33E-285 | 100.00 | 25.79 | 57.26 |

## Slide 5
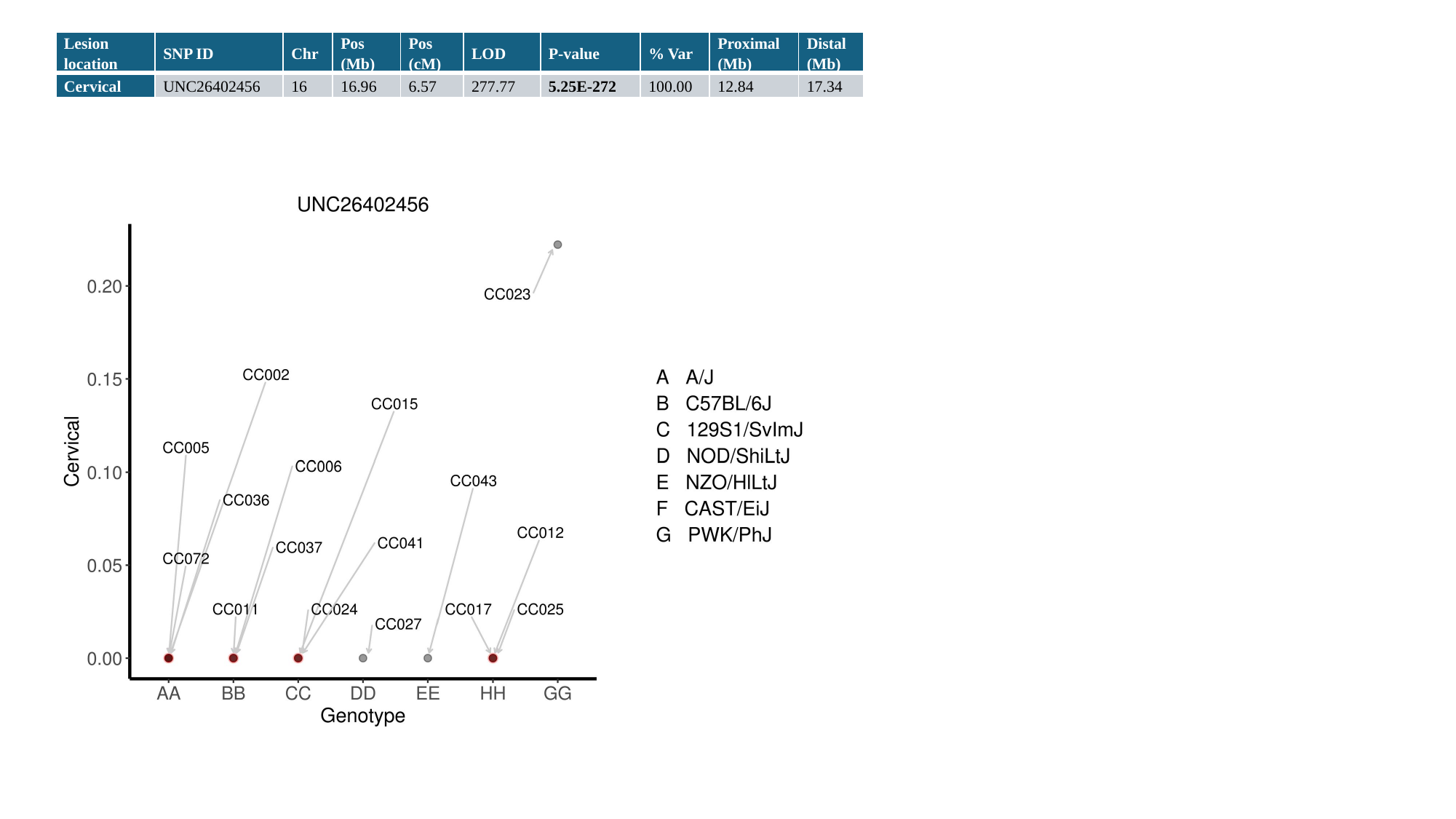

| Lesion location | SNP ID | Chr | Pos (Mb) | Pos (cM) | LOD | P-value | % Var | Proximal (Mb) | Distal (Mb) |
| --- | --- | --- | --- | --- | --- | --- | --- | --- | --- |
| Cervical | UNC26402456 | 16 | 16.96 | 6.57 | 277.77 | 5.25E-272 | 100.00 | 12.84 | 17.34 |

## Slide 6
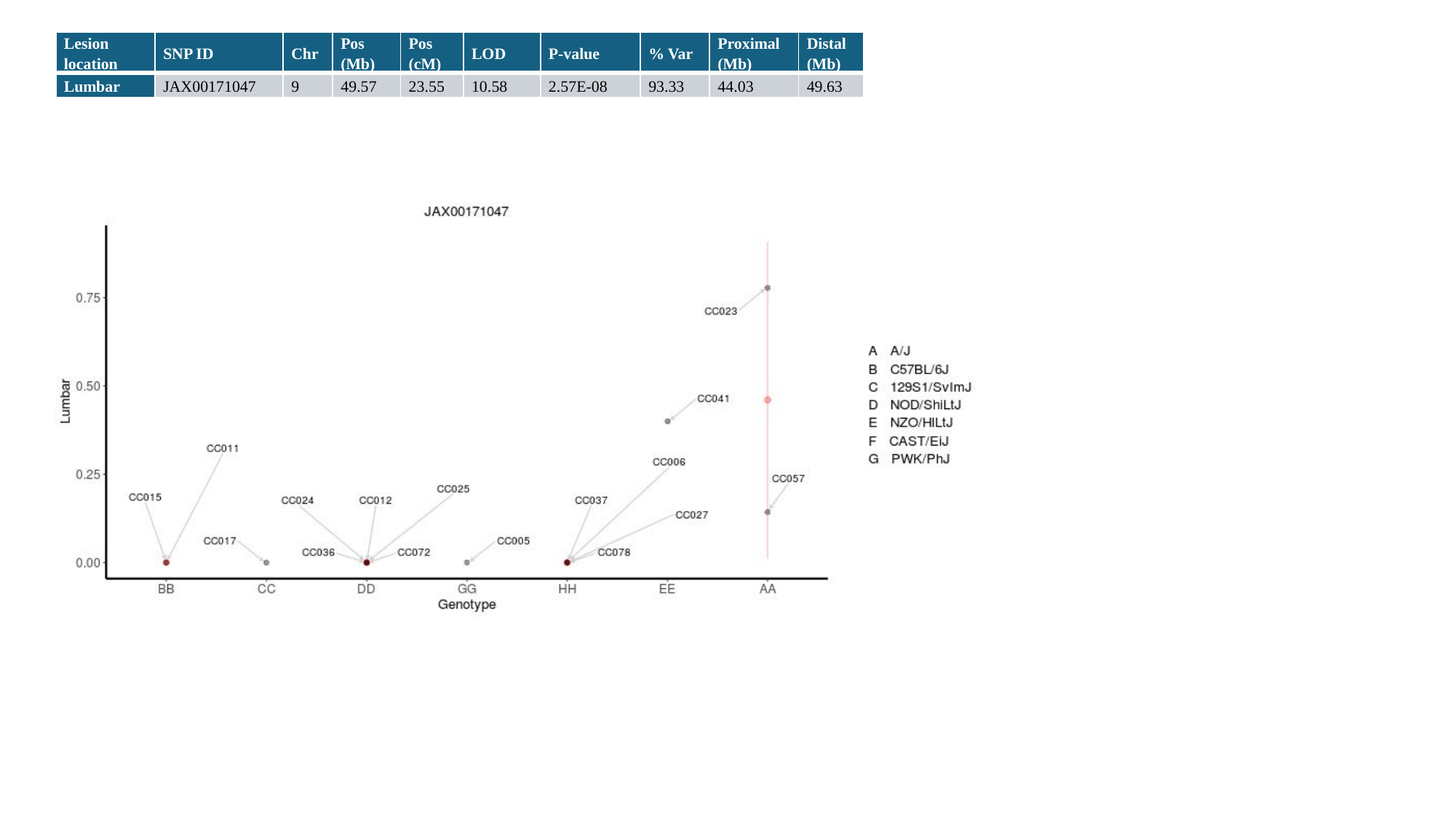

| Lesion location | SNP ID | Chr | Pos (Mb) | Pos (cM) | LOD | P-value | % Var | Proximal (Mb) | Distal (Mb) |
| --- | --- | --- | --- | --- | --- | --- | --- | --- | --- |
| Lumbar | JAX00171047 | 9 | 49.57 | 23.55 | 10.58 | 2.57E-08 | 93.33 | 44.03 | 49.63 |

## Slide 7
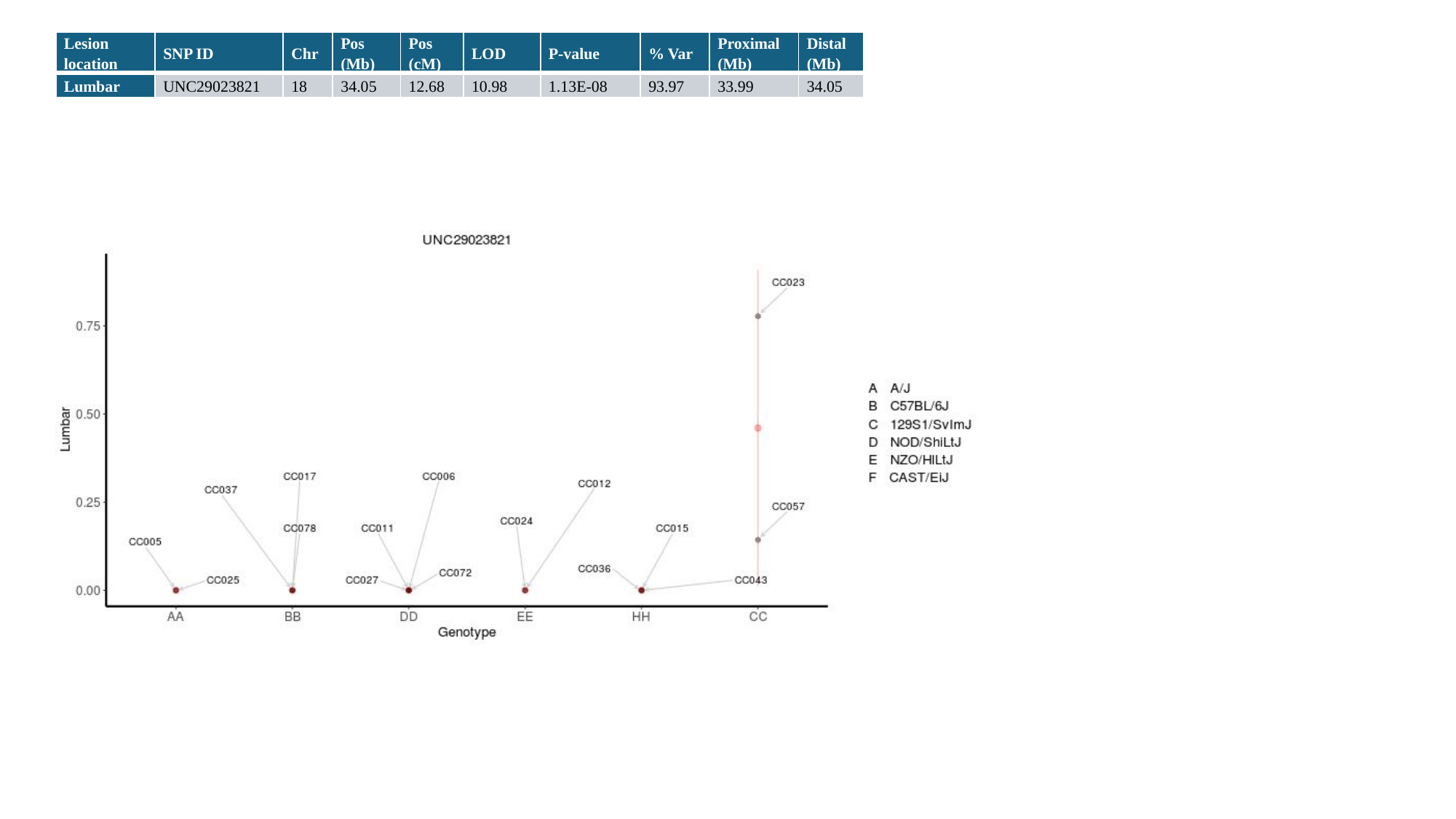

| Lesion location | SNP ID | Chr | Pos (Mb) | Pos (cM) | LOD | P-value | % Var | Proximal (Mb) | Distal (Mb) |
| --- | --- | --- | --- | --- | --- | --- | --- | --- | --- |
| Lumbar | UNC29023821 | 18 | 34.05 | 12.68 | 10.98 | 1.13E-08 | 93.97 | 33.99 | 34.05 |
